# Joint effects of ambient air pollution and maternal smoking on neonatal adiposity and childhood BMI trajectories in the Healthy Start study

**DOI:** 10.1097/EE9.0000000000000142

**Published:** 2021-05-05

**Authors:** Brianna F. Moore, Anne P. Starling, Sheena E. Martenies, Sheryl Magzamen, Dana Dabelea

**Affiliations:** a Department of Epidemiology, Human Genetics, and Environmental Sciences, The University of Texas Health Science Center, Austin, Texas; b Department of Epidemiology, Colorado School of Public Health, Aurora, Colorado; c Lifecourse Epidemiology of Adiposity and Diabetes (LEAD) Center, Colorado School of Public Health, Aurora, Colorado; d Department of Environmental and Radiological Health Sciences, Colorado State University, Fort Collins, Colorado; e Department of Pediatrics, University of Colorado School of Medicine, Aurora, Colorado.

**Keywords:** Interaction, Air pollution, Maternal smoking, Adiposity, Growth trajectories

## Abstract

Coexposure to air pollution and tobacco smoke may influence early-life growth, but few studies have investigated their joint effects. We examined the interaction between fetal exposure to maternal smoking and ozone (O_3_) or fine particulate matter (PM_2.5_) on birth weight, neonatal adiposity, and body mass index (BMI) trajectories through age 3 years.

**Methods:**

Participants were 526 mother-child pairs, born ≥37 weeks. Cotinine was measured at ~27 weeks gestation. Whole pregnancy and trimester-specific O_3_ and PM_2.5_ were estimated via. inverse-distance weighted interpolation from stationary monitors. Neonatal adiposity (fat mass percentage) was measured via. air displacement plethysmography. Child weight and length/height were abstracted from medical records. Interaction was assessed by introducing cotinine (<31.5 vs. ≥31.5 ng/mL [indicating active smoking]), O_3_/PM_2.5_ (low [tertiles 1–2] vs. high [tertile 3]), and their product term in linear regression models for birth weight and neonatal adiposity and mixed-effects models for BMI trajectories.

**Results:**

The rate of BMI growth among offspring jointly exposed to maternal smoking and high PM_2.5_ (between 8.1 and 12.7 μg/m^3^) in the third trimester was more rapid than would be expected due to the individual exposures alone (0.8 kg/m^2^ per square root year; 95% CI = 0.1, 1.5; *P* for interaction = 0.03). We did not detect interactions between maternal smoking and O_3_ or PM_2.5_ at any other time on birth weight, neonatal adiposity, or BMI trajectories.

**Conclusions:**

Although PM_2.5_ was generally below the EPA annual air quality standards of 12.0 μg/m^3^, exposure during the third trimester may influence BMI trajectories when combined with maternal smoking.

## Abstract 2

Supplemental Digital Content is available in the text.

**What This Study Adds**

Fetal exposure to maternal smoking and ambient air pollution has been linked to low birth weight followed by rapid growth in early childhood. However, little is known about their potential joint effects. Our interaction results suggest that exposure to relatively low levels of fine particulate matter ([PM_2.5_] between 8.1 and 12.7 μg/m^3^) during the third trimester contributes to rapid BMI growth during the first 3 years of life when combined with maternal smoking. Childhood obesity prevention strategies should encourage smoking cessation and the avoidance of exposure to PM_2.5_ among pregnant women to achieve the maximum public health benefit.

## Introduction

Low-birth weight followed by rapid weight gain in the first few years of life (a pattern known as catch-up growth) is widely accepted as an early predictor of obesity.^1^ This pattern of growth has been linked to many environmental pollutants, including tobacco smoke. For nearly 5 decades, maternal active smoking during pregnancy has been consistently linked to low birth weight.^2^ Additional research has since demonstrated that maternal smoking during pregnancy is associated with a reduction in neonatal adiposity^3,4^ followed by rapid body mass index (BMI) growth in early childhood.^4^

In utero exposure to other pollutants, such as ambient ozone (O_3_) and fine particulate pollution (PM_2.5_), may be associated with a similar pattern of growth. Studies have demonstrated that fetal exposures to these widespread pollutants are associated with low birth weight^5–7^ followed by rapid infant weight gain^8^ but not childhood BMI trajectories.^9^ In contrast to previous studies, results from our own cohort provide limited evidence of an independent association between fetal exposures to O_3_ or PM_2.5_ with birth weight.^10,11^ One possibility is that concurrent exposure to tobacco smoke may exacerbate the proinflammatory responses induced by exposure to ambient air pollution,^12–14^ contributing to atypical growth of the offspring.

Three population-based cohort studies have explored whether the association between fetal exposure to particulate air pollution and birth weight is stronger among active smokers but the evidence is mixed.^15–17^ The largest study, conducted among 231,929 mother-child pairs in British Columbia, provided evidence that the effect of PM_2.5_ on birth weight was stronger among mothers who actively smoked during pregnancy.^17^ However, no interaction was detected in the Japanese or European populations.^15,16^ In light of these inconsistent findings, there is a need to extend this analysis to other populations and to other pollutants, such as O_3_. Finally, since these exposures can have lasting effects on childhood BMI, there is a need to assess whether maternal smoking modifies the association between ambient air pollution and childhood BMI trajectories.

We aimed to assess the potential interaction between fetal exposure to maternal smoking and O_3_ or PM_2.5_ with body composition at birth and BMI growth trajectories through age 3 years. This analysis was conducted among mother-child pairs enrolled in the Healthy Start, a longitudinal prebirth cohort in Colorado. We hypothesized that offspring with both exposures will experience deficits in birth weight followed by rapid BMI growth in the first 3 years of life that is greater than would be expected due to the effects of the individual exposures alone.

## Methods

### Study population

The Healthy Start study recruited 1,410 pregnant women aged ≥16 years with singleton pregnancies before 24 weeks of gestation from the obstetrics clinics at the University of Colorado Hospital between 2010 and 2014. Participants completed two research visits in pregnancy (median 17 and 27 weeks of gestation) and at delivery (median 1 day postdelivery). Women were excluded from this study if they were expecting multiple births; had a previous stillbirth or preterm birth before 25 weeks of gestation; or had preexisting diabetes, asthma, cancer, or psychiatric illness. Mother-child pairs were eligible for the body composition analysis if they had complete data on body composition measures at birth and had cotinine measured in stored maternal urine samples. Mother-child pairs were additionally eligible for the childhood BMI analysis if they had reached the age of 3 years by July 2019, had ≥3 weight and length/height measurements from pediatric visits, and had cotinine measured in stored maternal urine samples. The Healthy Start study protocol was approved by the Colorado Multiple Institutional Review Board. All women provided written informed consent before the first study visit. The Healthy Start study was registered as an observational study at clinicaltrials.gov as NCT02273297.

### Maternal urinary cotinine

Cotinine was measured in a subsample of women with stored urine samples collected at ~27 weeks gestation. Cotinine was measured via. solid phase competitive ELISA, with a sensitivity of 1 ng/mL (Calbiotech Cotinine ELISA CO096D, Calbiotech, El Cajon, California). The limit of detection (LOD) was 0.05 ng/mL. We categorized women as either nonsmoker (cotinine < 31.5 ng/mL; the established cutpoint for active smoking^18^) or active smoker (≥31.5 ng/mL).

### Air pollutant data

Ambient O_3_ and PM_2.5_ concentrations were obtained from the US Environmental Protection Agency (EPA) Air Quality System (AQS) Data Mart Information (https://www3.epa.gov/airdata/) and from the Colorado Department of Public Health and Environment. Average ozone concentrations (ppm) were generally measured every hour. Hourly values were averaged over 8-hour intervals during a 24-hour period. Daily 8-hour maximum values of O_3_ were used in this analysis. Average daily concentrations of PM_2.5_ (μg/m^3^) were measured every 1–6 days, although most were measured every 3 or 6 days. Average daily exposures for the duration of each pregnancy were assigned to individual mothers based on the conception dates and the first known address, as previously described by Starling and colleagues.^11^ Briefly, an inverse distance weighting approach was employed in which the average values of all available monitors with 50 km of the participant were weighted according to the formula 1/distance-squared. Average daily exposures for each participant were derived for each trimester and for the entire pregnancy.

### Neonatal body composition

Fat mass and fat-free mass were measured within ~72 hours of delivery by trained study staff using whole body air displacement plethysmography (PEA POD, COSMED, Rome, Italy). The PEA POD system measures body mass and volume, calculates body density, and estimates fat mass (g) and fat-free mass (g). Fat mass and fat-free mass were measured twice. If the percent fat mass differed by more than 2.0%, a third measurement was taken. The average of the two closest readings was used in this analysis. Percent fat mass was calculated as fat mass divided by the sum of fat mass and fat-free mass. Birth weight was obtained from obstetric records.

### Child BMI

We abstracted weight, recumbent length (generally until 24 months), and standing height (generally after 24 months) from medical records at pediatric visits. These measurements were generally recorded at well-child visits, which occur at 1, 2, 4, 6, 12, 18, 24, 30, and 36 months. BMI was calculated by dividing weight in kilograms by height in meters squared.

### Covariates

Mother and child characteristics were collected during the research visits and through medical records. Maternal age at delivery was calculated by subtracting the participant’s date of birth from the date of delivery. Maternal race/ethnicity, maternal education, and annual household income were self-reported via. study questionnaires. Maternal height was measured using a stadiometer during the first pregnancy research visit. Prepregnancy weight was obtained from medical records (91%) or self-reported at the first pregnancy research visit (9%). Prepregnancy BMI was calculated as prepregnancy weight (kg) divided by height squared (m^2^). Gestational weight gain was calculated as the difference between the last available weight measurement during pregnancy (measured by research staff or medical personnel) and prepregnancy weight. The mean gestational age at the last available weight measurement was 38.2 weeks. Census tract-level socioeconomic data were obtained from the 2012 to 2016 American Community Survey. The median income and percentage of persons below the poverty level were linked to individual participant addresses within a given Census tract in ArcGIS Desktop 10.X, as previously described.^11^

Mothers were asked to report the number of adults in the household (including themselves) who were regular smokers at 5 months and 18 months of age. Responses to this question ranged from zero to six. We dichotomized these data into no household smokers and any household smokers (if they indicated at least one household smoker at 5 months or 18 months of age).

The duration of breastfeeding exclusivity was ascertained via. questionnaire at age 5 months. Women were asked if they were currently feeding their infant any breast milk, had ever fed their infant formula, or were currently feeding their infant formula. The duration of breastfeeding exclusivity variable was dichotomized as <5 months and ≥5 months.

### Statistical analysis

Separate linear regression models estimated the interaction between the cotinine categories (nonsmoker versus smoker) and O_3_ or PM_2.5_ (low versus high) on birth weight (g) and percent fat mass at birth. We modeled O_3_ and PM_2.5_ for each trimester separately, since the influence of these exposures on birth outcomes and postnatal growth may differ across various stages of gestation.^5,8,19^ We used the Akaike information criteria (AIC) and Bayesian information criteria (BIC) values to determine the best-fitting interaction models, where lower values represent a better-fitting model. We compared continuous and categorized assessments (median-split, tertiles, quartiles) of O_3_ or PM_2.5_. The lowest AIC and BIC values for the interaction models were achieved when O_3_ or PM_2.5_ was dichotomized as low (first and second tertiles) or high exposure (the third tertile). Interaction was evaluated by including product terms between the dichotomous cotinine and O_3_/PM_2.5_ variables in separate models. We adjusted for confounders that are related to maternal smoking during pregnancy, exposure to ambient air pollution, and birth weight/adiposity, including maternal age (years), gestational weight gain (kg), prepregnancy BMI (kg/m^2^), maternal race/ethnicity (nonHispanic white, nonHispanic Black, Hispanic, other), maternal education (<high school, high school diploma, any college), offspring sex, gestational age at birth (weeks), season of birth (spring, summer, fall, winter), year of birth, and median household income by Census tract (quartiles).

Mixed-effects regression models estimated the longitudinal association between the dichotomous cotinine and O_3_ or PM_2.5_ variables with BMI levels through age 3 years. Mixed-effects models allow for repeated measures and can be applied when outcome data are measured at different time points or are sparsely measured over time. Based on the deviance information criteria,^20^ the best-fit trajectory for the age was a square root transformation. Assumptions of linearity and homoscedasticity were verified via. examination of the jackknifed-studentized residuals. We used Wald tests with Kenward-Roger degrees of freedom.^21^ In addition to the covariates above, we adjusted for self-report of household smokers in early childhood (none, any) and the duration of exclusive breastfeeding (<5 months, ≥5 months).

All statistical analyses were conducted using Stata, Version 14.2 (StataCorp LP, College Station, TX). An alpha level of 0.05 was used to determine statistical significance of the interaction analyses.

### Sensitivity analyses

The published literature has examined the association between prenatal exposure to air pollution or maternal smoking and childhood growth trajectories using both absolute BMI values^9,22–25^ and BMI z-scores.^26–28^ As a sensitivity analysis, we also performed the mixed-effects models with BMI z-score trajectories as the outcome of interest. Age of the child was treated as a continuous variable (years), based on the slope of the BMI z-score trajectories and the deviance information criteria.^20^

## Results

Of the 1,410 participants enrolled in the Healthy Start cohort study, 1,338 children were born at or after 37 weeks gestation. Of these, 691 mother-child pairs had cotinine measured in stored urine samples from mid-pregnancy. Of these, 72 mother-child pairs were missing complete body composition measures at birth, 39 were missing full-pregnancy estimates of PM_2.5_, and 6 were missing data on gestational weight gain. Therefore, the final sample size for the body composition analyses was 575 mother-child pairs. For the analyses of BMI growth trajectories, we further excluded 66 mother-child pairs who did not have at least three length/height and weight measurements abstracted from medical records as of October 2017. The final sample size for the childhood BMI analyses was 434, due to missing information regarding postnatal exposure to secondhand smoke and the duration of exclusive breastfeeding. There were no substantial differences in maternal or child characteristics for the analytic samples compared with the entire cohort (eTable 1; http://links.lww.com/EE/A137).

Maternal and child characteristics are presented in Table 1. Based on maternal urinary cotinine, 61 women (11%) were classified as active smokers and 514 women (89%) were classified as nonsmokers. Compared with active smokers, women classified as nonsmokers were older (*P* < 0.01) and reported less pregnancies (*P* < 0.01). Nonsmokers were more likely to be non-Hispanic White (*P* < 0.01), to have attended college (*P* < 0.01), and to have an annual household income above $70,000 (*P* < 0.01). Offspring born to nonsmokers were more likely to have been breastfed exclusively until age 5 months (*P* < 0.01) but less likely to significantly more likely to live with a household smoker at age 5 months (*P* < 0.01). There were no differences in prepregnancy BMI (*P* = 0.26), gestational weight gain (*P* = 0.48), and offspring sex (*P* = 0.08).

**Table 1.** Characteristics of eligible mother-child pairs in the Healthy Start study, according to cotinine categories.

		Prenatal cotinine categories^a^		
	All (n = 575)	Nonsmoker (n = 514)	Active smoking (n = 61)	*P*
**Mother characteristics**				
Age (years)	29 ± 6	29 ± 6	26 ± 5	<0.01
Prepregnancy body mass index (kg/m^2^)	25 ± 6	25 ± 6	26 ± 7	0.26
Gestational weight gain (kg)	14 ± 6	14 ± 6	14 ± 8	0.48
Previous pregnancies (any)	1 ± 1	1 ± 1	2 ± 2	<0.01
Race/ethnicity				
Non-Hispanic White	55%	55%	37%	
Non-Hispanic Black	12%	10%	40%	
Hispanic	28%	29%	17%	
Other	5%	6%	7%	<0.01
Highest level of education				
<High school	15%	11%	30%	
High school degree	15%	15%	25%	
Some college or more	70%	74%	45%	<0.01
Household income				
<$40,000	26%	23%	47%	
$40,001 to $70,000	13%	19%	20%	
>$70,000	39%	40%	8%	
Do not know	21%	18%	25%	<0.01
Median income in Census tract (in $1000s)	64 ± 28	67 ± 30	55 ± 21	0.01
**Child characteristics**				
Male	52%	49%	62%	0.08
Gestational age at birth (weeks)	40 ± 1	40 ± 1	39 ± 1	<0.01
Birthweight (g)	3,309 ± 427	3,345 ± 416	3,009 ± 409	<0.01
Neonatal adiposity (% fat mass)	9.1 ± 3.9	9.2 ± 3.9	8.2 ± 3.6	0.03
Household smokers during early childhood, n = 445				
None	85%	91%	37%	
Any	15%	9%	64%	<0.01
Duration of exclusive breastfeeding, n = 461				
<5 months	53%	49%	91%	
≥5 months	47%	51%	9%	<0.01
**Ambient exposures during pregnancy**				
Trimester 1 average PM_2.5_ (μg/m^3^), n = 479	7.6 ± 0.8	7.5 ± 0.8	7.4 ± 0.7	0.18
Tertile 1 (5.5–7.2 μg/m^3^)		32%	41%	
Tertile 2 (7.2–7.9 μg/m^3^)		34%	30%	
Tertile 3 (7.9–10.7 μg/m^3^)		34%	29%	0.42
Trimester 2 average PM_2.5_ (μg/m^3^), n = 477	7.6 ± 0.9	7.6 ± 1.0	7.6 ± 0.7	0.99
Tertile 1 (5.1–7.2 μg/m^3^)		33%	37%	
Tertile 2 (7.2–8.0 μg/m^3^)		33%	35%	
Tertile 3 (8.0–10.8 μg/m^3^)		34%	28%	0.66
Trimester 3 average PM_2.5_ (μg/m^3^), n = 510	7.6 ± 1.1	7.6 ± 1.1	7.7 ± 1.1	0.78
Tertile 1 (5.1–7.1 μg/m^3^)		33%	35%	
Tertile 2 (7.1–8.1 μg/m^3^)		34%	27%	
Tertile 3 (8.1–12.7 μg/m^3^)		33%	38%	0.56
Whole pregnancy average PM_2.5_ (μg/m^3^)	7.6 ± 0.4	7.6 ± 0.4	7.6 ± 0.4	0.66
Tertile 1 (6.4–7.4 μg/m^3^)		33%	37%	
Tertile 2 (7.4–7.7 μg/m^3^)		35%	25%	
Tertile 3 (7.7–9.4 μg/m^3^)		33%	38%	0.32
Trimester 1 average 8-hour max O_3_ (ppb)	43.9 ± 11.1	43.6 ± 11.1	46.8 ± 10.5	0.03
Tertile 1 (20.1–35.9 ppb)		35%	25%	
Tertile 2 (35.9–51.2 ppb)		33%	30%	
Tertile 3 (51.2–62.4 ppb)		32%	45%	0.10
Trimester 2 average 8-hour max O_3_ (ppb)	42.4 ± 10.6	42.4 ± 10.6	42.2 ± 10.6	0.91
Tertile 1 (20.1–34.8 ppb)		33%	37%	
Tertile 2 (34.8–48.2 ppb)		34%	30%	
Tertile 3 (48.2–62.3 ppb)		33%	33%	0.80
Trimester 3 average 8-hour max O_3_ (ppb)	43.2 ± 10.5	43.5 ± 10.5	41.1 ± 10.2	0.10
Tertile 1 (23.0–35.9 ppb)		32%	42%	
Tertile 2 (35.9–50.1 ppb)		34%	30%	
Tertile 3 (50.1–61.2 ppb)		34%	28%	0.33
Whole pregnancy average 8-hour max O_3_ (ppb)	43.3 ± 4.0	43.3 ± 4.0	43.5 ± 3.6	0.68
Tertile 1 (28.9–41.7 ppb)		34%	30%	
Tertile 2 (41.7–45.2 ppb)		32%	35%	
Tertile 3 (45.2–52.9 ppb)		34%	35%	0.81

Continuous variables are expressed as means ± standard deviation. Independent samples t-tests were used to examine the differences in means by cotinine categories. Categorical variables are expressed as proportions of column totals. Chi-square tests were used to examine differences in proportions by cotinine categories.

^a^The cotinine categories were defined as follows: nonsmoker (<31.5 ng/mL) or active smoker (≥31.5 ng/mL).

O_3_ indicates ozone; PM_2.5_, fine particulate matter.

We did not detect an interaction between fetal exposure to maternal smoking with PM_2.5_ on birth weight or neonatal adiposity (Table 2). There was some indication that the association between high exposure to PM_2.5_ during the third trimester and neonatal adiposity varies by smoking status of the mother. Within the stratum of active smokers, high exposure to PM_2.5_ during third trimester was associated with decreased neonatal adiposity (beta coefficient: –3.5%; 95% CI = –7.0%, –0.1%). Conversely, within the stratum of nonsmokers, there was virtually no difference in neonatal adiposity between those with low and high exposure to PM_2.5_ during the third trimester (beta coefficient: –0.3%; 95% CI = –1.2%, 0.6%).

**Table 2.** Adjusted means and mean differences of neonatal body composition in relation to fetal exposure to maternal smoking and PM_2.5_ exposure by trimester^a^.

		**Birth weight (g**)			**Neonatal adiposity (% fat mass**)		
Cotinine categories^b^	PM_2.5_ categories^c^	n	Adjusted mean among offspring born to nonsmoker with low PM_2.5_ exposure and mean differences (CIs)	Stratified beta coefficients	n	Adjusted mean among offspring born to nonsmoker with low PM_2.5_ exposure and mean differences (CIs)	Stratified beta coefficients
		Whole pregnancy			Whole pregnancy		
Nonsmoker	Low	346	3,320 (3,275, 3,365)	Reference	346	9.1 (8.7, 9.6)	Reference
	High	168	50 (–32, 131)	51 (–32, 134)	168	–0.1 (–0.9, 0.7)	–0.1 (–0.9, 0.7)
Smoker	Low	37	–233 (–375, –91)	Reference	37	–0.6 (–2.0, 0.8)	Reference
	High	24	–351 (–529, –174)	–188 (–436, 59)	24	–1.9 (–3.6, –0.2)	–1.4 (–4.1, 1.4)
*P* for interaction			*P* = 0.14			*P* = 0.27	
		Trimester 1			Trimester 1		
Nonsmoker	Low	308	3,344 (3,294, 3,393)	Reference	308	9.3 (8.8, 9.8)	Reference
	High	161	–8 (–100, 84)	–12 (–107, 83)	161	–0.4 (–1.3, 0.5)	–0.5 (–1.4, 0.4)
Smoker	Low	40	–300 (–442, –158)	Reference	40	–0.8 (–2.2, 0.6)	Reference
	High	16	–214 (–425, –4)	9 (–281, 298)	16	–1.5 (–3.5, 0.6)	0.2 (–3.0, 3.4)
*P* for interaction			*P* = 0.42			*P* = 0.81	
		Trimester 2			Trimester 2		
Nonsmoker	Low	306	3,334 (3,284, 3,384)	Reference	306	9.3 (8.8, 9.8)	Reference
	High	155	–15 (–109, 78)	–22 (–119, 75)	155	–0.4 (–1.4, 0.5)	–0.3 (–1.2, 0.6)
Smoker	Low	40	–249 (–390, –108)	Reference	40	–1.0 (–2.4, 0.4)	Reference
	High	18	–307 (–517, –98)	23 (–308, 355)	18	–1.4 (–3.5, 0.6)	–0.2 (–3.7, 3.4)
*P* for interaction			*P* = 0.73			*P* = 0.99	
		Trimester 3			Trimester 3		
Nonsmoker	Low	338	3,341 (3,293, 3,388)	Reference	338	9.2 (8.7, 9.6)	Reference
	High	163	–17 (–107, 73)	–7 (–100, 85)	163	–0.2 (–1.0, 0.6)	–0.3 (–1.2, 0.6)
Smoker	Low	34	–280 (–431, –128)	Reference	34	–0.6 (–2.5, 1.3)	Reference
	High	22	–307 (–497, –116)	–116 (–401, 169)	22	–1.5 (–3.0, –0.1)	–3.5 (–7.0, –0.1)
*P* for interaction			*P* = 0.93			*P* = 0.67	

^a^All models adjusted for offspring sex, gestational age at birth (weeks), maternal prepregnancy BMI (kg/m^2^), gestational weight gain (kg), maternal education (high school, some college, college), maternal race/ethnicity (non-Hispanic White, non-Hispanic Black, Hispanic, other), annual household income (<$40,000, $40,001 to $70,000, >$70,000, missing or do not know), temperature (F), birth year (2010, 2011, 2012, 2013, 2014), season of birth (spring, summer, fall, winter), and median household income by Census tract (in $1000s).

^b^The cotinine categories were defined as follows: nonsmoker (<31.5 ng/mL) or active smoker (≥31.5 ng/mL).

^c^The PM_2.5_ categories were defined as follows: low (first and second tertile of PM_2.5_) and high (third tertile of PM_2.5_).

^d^Additionally adjusted for infant age in days at follow-up and the duration of exclusive breastfeeding (<5 months, ≥5 months).

CI indicates confidence interval; PM_2.5_, fine particulate matter.

Similar to the PM_2.5_ results, the interaction results do not support the hypothesis that fetal exposure to maternal smoking and O_3_ act synergistically to influence birth weight or neonatal adiposity (Table 3). There were no indications that the associations between O_3_ and birth weight or neonatal adiposity were stronger within the stratum of offspring born to active smoking mothers.

**Table 3.** Adjusted means and mean differences of neonatal body composition in relation to fetal exposure to maternal smoking and O_3_ exposure by trimester^a^.

		**Birth weight (g**)			**Neonatal adiposity (% fat mass**)		
Cotinine categories^b^	O categories^c^	n	Adjusted means among offspring born to nonsmoker with low O_3_ exposure and mean differences (CIs)	Stratified beta coefficients	n	Adjusted means among offspring born to nonsmoker with low O_3_ exposure and mean differences (CIs)	Stratified beta coefficients
		Whole pregnancy			Whole pregnancy		
Nonsmoker	Low	343	3,337 (3,285, 3,389)	Reference	343	9.0 (8.5, 9.5)	Reference
	High	175	–3 (–113, 108)	–27 (–142, 88)	175	0.3 (–0.8, 1.3)	0.2 (–0.9, 1.3)
Smoker	Low	40	–299 (–439, –159)	Reference	40	–1.2 (–2.5, 0.2)	Reference
	High	21	–284 (–489, –80)	100 (–319, 518)	21	–0.6 (–2.6, 1.4)	0.2 (–4.4, 4.8)
*P* for interaction			*P* = 0.88			*P* = 0.79	
		Trimester 1			Trimester 1		
Nonsmoker	Low	353	3,336 (3,278, 3,395)	Reference	353	8.9 (8.3, 9.4)	Reference
	High	165	–5 (–144, 134)	–13 (–160, 134)	165	0.7 (–0.6, 2.0)	0.4 (–0.9, 1.8)
Smoker	Low	34	–327 (–479, –175)	Reference	34	–1.3 (–2.7, 0.2)	Reference
	High	27	–228 (–433, –23)	113 (–403, 629)	27	–0.2 (–2.2, 1.8)	0.9 (–4.6, 6.5)
*P* for interaction			*P* = 0.36			*P* = 0.76	
		Trimester 2			Trimester 2		
Nonsmoker	Low	346	3,332 (3,270, 3,394)	Reference	346	9.3 (8.7, 9.9)	Reference
	High	172	12 (–137, 161)	0 (–157, 157)	172	–0.5 (–1.9, 1.0)	–0.6 (–2.0, 0.9)
Smoker	Low	39	–264 (–401, –127)	Reference	39	–1.0 (–2.3, 0.3)	Reference
	High	22	–345 (–578, –112)	–152 (–707, 403)	22	–1.7 (–4.0, 0.5)	–1.4 (–7.5, 4.7)
*P* for interaction			*P* = 0.42			*P* = 0.80	
		Trimester 3			Trimester 3		
Nonsmoker	Low	341	3,339 (3,269, 3,389)	Reference	341	9.0 (8.4, 9.6)	Reference
	High	177	18 (–125, 160)	5 (–144, 154)	177	0.4 (–1.0, 1.7)	0.5 (–0.9, 2.0)
Smoker	Low	43	–284 (–419, –149)	Reference	43	–0.9 (–2.2, 0.4)	Reference
	High	18	–264 (–485, –43)	172 (–281, 626)	18	–1.3 (–3.4, 0.9)	0 (–4.9, 4.9)
*P* for interaction			*P* = 0.98			*P* = 0.53	

^a^All models adjusted for offspring sex, gestational age at birth (weeks), maternal prepregnancy BMI (kg/m^2^), gestational weight gain (kg), maternal education (high school, some college, college), maternal race/ethnicity (non-Hispanic White, non-Hispanic Black, Hispanic, other), annual household income (<$40,000, $40,001 to $70,000, >$70,000, missing or do not know), temperature (F), birth year (2010, 2011, 2012, 2013, 2014), season of birth (spring, summer, fall, winter), and median household income by Census tract (in $1000s).

^b^The cotinine categories were defined as follows: nonsmoker (<31.5 ng/mL) or active smoker (≥31.5 ng/mL).

^c^The O_3_ categories were defined as follows: low (first and second tertile of O_3_) and high (third tertile of O_3_).

^d^Additionally adjusted for infant age in days at follow-up and the duration of exclusive breastfeeding (<5 months, ≥5 months).

CI indicates confidence interval; O_3_, ozone.

**Figure 1.**
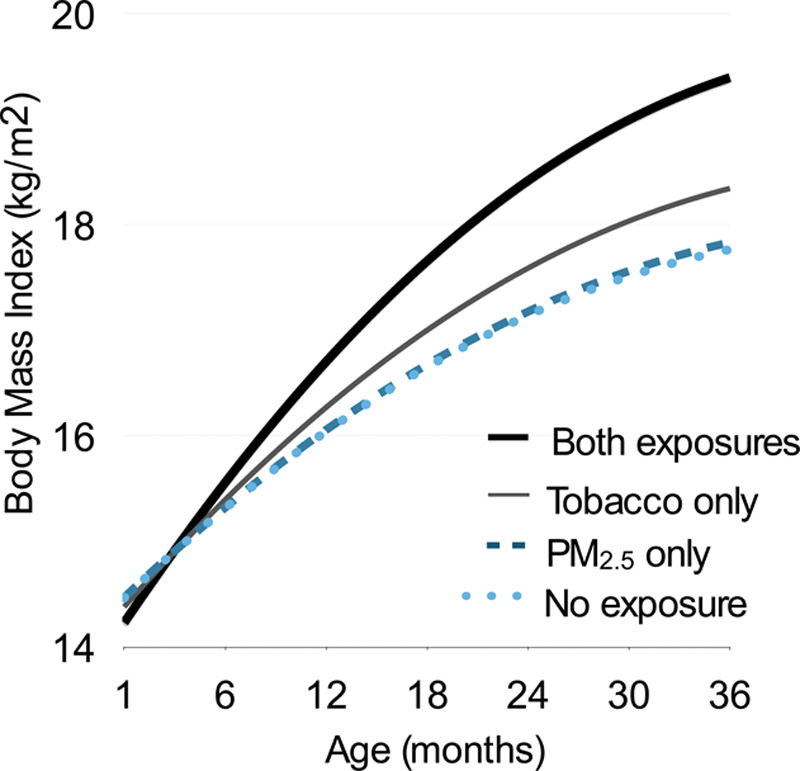
Childhood BMI trajectories according to fetal exposure to maternal smoking during pregnancy and exposure to PM_2.5_ in the third trimester. Exposure categories were defined as follows: no exposure (low PM_2.5_ [between 5.1 and 8.1 μg/m^3^] and cotinine <31.5); high PM_2.5_ only (high PM_2.5_ [between 8.1 and 12.7 μg/m^3^] and cotinine<31.5); maternal smoking only (low PM_2.5_ and cotinine ≥31.5 ng/mL); and both exposures (high PM_2.5_ and cotinine ≥31.5 ng/mL).The mixed-effects model adjusted for offspring sex, gestational age at birth (weeks), maternal prepregnancy BMI (kg/m^2^), gestational weight gain (kg), maternal education (high school, some college, college), maternal race/ethnicity (non-Hispanic White, non-Hispanic Black, Hispanic, other), annual household income (<$40,000, $40,001 to $70,000, >$70,000, missing or do not know), temperature (F), birth year (2010, 2011, 2012, 2013, 2014), season of birth (spring, summer, fall, winter), household smokers in early childhood (any, none), the duration of exclusive breastfeeding (<5 months, ≥5 months), and median household income by Census-tract (in $1000s). The rate of BMI growth among offspring exposed to maternal smoking and high PM_2.5_ in the third trimester (PM_2.5_ between 8.1 and 12.7 μg/m^3^) was more rapid than would be expected due to the individual exposures alone (0.8 kg/m^2^ per square root year; 95% CI = 0.1, 1.5; *P* for interaction = 0.03). BMI indicates body mass index.

Table 4 shows the results for the interaction between fetal exposure to maternal smoking and PM_2.5_ on childhood BMI trajectories. We detected a statistically significant interaction between fetal exposure to maternal smoking, fetal exposure to high PM_2.5_ during the third trimester, and age on childhood BMI trajectories (*P* = 0.03). Compared with offspring with no exposure to maternal smoking and low PM_2.5_ exposure during the third trimester, BMI growth was 0.8 kg/m^2^ higher per square root year (95% CI = 0.1, 1.5) among offspring with both exposures, whereas BMI growth was only 0.4 kg/m^2^ higher (95% CI = 0.1, 0.8) among offspring with exposure to maternal smoking only and 0 kg/m^2^ higher (95% CI = –0.2, 0.2) among offspring with high PM_2.5_ exposure only. Figure 1 further illustrates the comparatively more rapid growth among offspring born to smoking mothers with high third trimester PM_2.5_ exposure, as compared to the other exposure levels. By age 3 years, the predicted BMI was 19.5 kg/m^2^ (95% CI = 18.6, 20.4) among offspring with exposure to maternal smoking and high PM_2.5_ exposure (eTable 2; http://links.lww.com/EE/A137). Predicted BMI levels were lower among offspring with exposure to maternal smoking only (18.4 kg/m^2^; 95% CI = 17.8, 19.0), offspring with high PM_2.5_ exposure only (17.9 kg/m^2^; 95% CI = 17.6, 18.1), and offspring with no exposure to maternal smoking and low PM_2.5_ exposure (17.8 kg/m^2^; 95% CI = 17.6, 18.0).

**Table 4.** Adjusted beta coefficients and 95% CIs for the association between fetal exposure to maternal smoking and PM_2.5_ with childhood BMI trajectories.

Covariates	Whole pregnancy	Trimester 1	Trimester 2	Trimester 3
Cotinine (smoker versus nonsmoker)	–0.1 (–0.7, 0.4)	–0.3 (–0.8, 0.3)	–0.1 (–0.8, 0.5)	–0.2 (–0.7, 0.3)
PM_2.5_ (high versus low)	0.1 (–0.2, 0.4)	0.2 (–0.1, 0.4)	0 (–0.3, 0.3)	0 (–0.3, 0.3)
Age (square root years)	2.3 (2.2, 2.5)	2.4 (2.2, 2.5)	2.4 (2.2, 2.6)	2.4 (2.3, 2.5)
Cotinine*PM_2.5_	–0.3 (–1.0, 0.4)	0 (–0.8, 0.7)	–0.3 (–1.0, 0.5)	–0.4 (–1.2, 0.4)
Cotinine*Age	0.2 (0, 0.8)	0.7 (0.2, 1.1)	0.7 (0.2, 1.2)	0.4 (0.1, 0.8)
PM_2.5_*Age	0.1 (–0.1, 0.)	0 (–0.2, 0.2)	0 (–0.2, 0.1)	0 (–0.2, 0.2)
Cotinine*PM_2.5_*Age	0.3 (–0.1, 1.2)	0.1 (–0.5, 0.7)	0 (–0.6, 0.6)	0.8 (0.1, 1.5)
*P* for three-way interaction	*P* = 0.51	*P* = 0.82	*P* = 0.53	*P* = 0.03

^a^All models adjusted for offspring sex, gestational age at birth (weeks), maternal prepregnancy BMI (kg/m^2^), gestational weight gain (kg), maternal education (high school, some college, college), maternal race/ethnicity (non-Hispanic White, non-Hispanic Black, Hispanic, other), annual household income (<$40,000, $40,001 to $70,000, >$70,000, missing or do not know), temperature (F), birth year (2010, 2011, 2012, 2013, 2014), season of birth (spring, summer, fall, winter), median household income by Census tract (in $1,000s), household smokers in early childhood (any, none), and the duration exclusive breastfeeding (<5 months, ≥5 months).

^b^The cotinine categories were defined as follows: nonsmoker (<31.5 ng/mL) or active smoker (≥31.5 ng/mL).

^c^The PM_2.5_ categories were defined as follows: low (first and second tertile of PM_2.5_) and high (third tertile of PM_2.5_).

BMI indicates body mass index; CI, confidence interval; PM_2.5_, fine particulate matter.

By contrast, the interaction results do not support the hypothesis that fetal exposure to maternal smoking and O_3_ act synergistically to influence childhood BMI trajectories (Table 5).

**Table 5.** Adjusted beta coefficients and 95% CIs for the association between fetal exposure to maternal smoking and O_3_ with childhood BMI trajectories.

Covariates	Whole pregnancy	Trimester 1	Trimester 2	Trimester 3
Cotinine (smoker versus nonsmoker)	0 (–0.7, 0.6)	0.1 (–0.6, 0.9)	–0.2 (–0.8, 0.4)	–0.1 (–0.7, 0.5)
O_3_ (high versus low)	–0.1 (–0.4, 0.1)	0.1 (–0.3, 0.5)	–0.1 (–0.5, 0.2)	–0.1 (–0.5, 0.2)
Age (square root years)	2.3 (2.1, 2.5)	2.4 (2.3, 2.6)	2.3 (2.1, 2.4)	2.3 (2.1, 2.4)
Cotinine*O_3_	–0.4 (–1.2, 0.3)	–0.6 (–1.4, 0.2)	–0.2 (–1.0, 0.5)	–0.4 (–1.1, 0.3)
Cotinine*Age	0.5 (0, 1.1)	0.4 (–0.2, 1.1)	0.7 (0.2, 1.1)	0.5 (0.1, 0.9)
O_3_*Age	0.1 (–0.1, 0.3)	–0.1 (–0.3, 0.1)	0.1 (–0.1, 0.3)	0.1 (–0.1, 0.3)
Cotinine*O_3_*Age	0.3 (–0.4, 0.9)	0.4 (–0.3, 0.1)	0.1 (–0.5, 0.8)	0.4 (–0.1, 1.1)
*P* for three-way interaction	*P* = 0.39	*P* = 0.87	*P* = 0.89	*P* = 0.42

^a^All models adjusted for offspring sex, gestational age at birth (weeks), maternal prepregnancy BMI (kg/m^2^), gestational weight gain (kg), maternal education (high school, some college, college), maternal race/ethnicity (non-Hispanic White, non-Hispanic Black, Hispanic, other), annual household income (<$40,000, $40,001 to $70,000, >$70,000, missing or do not know), temperature (F), birth year (2010, 2011, 2012, 2013, 2014), season of birth (spring, summer, fall, winter), median household income by Census tract (in $1,000s), household smokers in early childhood (any, none), and the duration exclusive breastfeeding (<5 months, ≥5 months).

^b^The cotinine categories were defined as follows: nonsmoker (<31.5 ng/mL) or active smoker (≥31.5 ng/mL).

^c^The O_3_ categories were defined as follows: low (first and second tertile of O_3_) and high (third tertile of O_3_).

BMI indicates body mass index; CI, confidence interval; O_3_, ozone.

### Sensitivity analyses

Our results tended to agree when we used BMI z-scores as the outcome of interest. However, the interaction between fetal exposure to maternal smoking and PM_2.5_ in the third trimester on childhood BMI z-score trajectories was slightly attenuated (eTable 3; http://links.lww.com/EE/A137; *P* for interaction = 0.09). The interaction results do not support the hypothesis that fetal exposure to maternal smoking and O_3_ act synergistically to influence childhood BMI z-score trajectories (eTable 4; http://links.lww.com/EE/A137).

## Discussion

Among mothers who actively smoked during pregnancy, higher exposure to PM_2.5_ (greater than or equal to 8.1 and less than or equal to 12.7 μg/m^3^ [the maximum value]) in the third trimester was associated with rapid BMI growth in the first 3 years of life, but not birth weight or neonatal adiposity. Rapid BMI growth in early childhood, regardless of birth size, is an important early predictor of obesity in later life.^29^ Thus, childhood obesity prevention strategies should aim to reduce individual exposure to PM_2.5_ and encourage smoking cessation among pregnant women to achieve the maximum public health benefit.

Until recently, epidemiologic studies have primarily focused on the adverse health effects of single-pollutant exposures. However, many populations are concurrently exposed to several air pollutants, rather than a single exposure. Coexposure to secondhand smoke and ambient air pollution may act in a cumulative fashion to increase the risk for adverse health outcomes in children. For instance, research has demonstrated a synergistic effect between exposure to secondhand smoke and ambient particulate pollution on childhood asthma,^22^ wheeze,^23^ and other respiratory outcomes.^24,25^ Coexposure to maternal smoking and ambient particulate pollution may also influence early-life growth, but few studies have investigated the potential joint effects.

Our interaction results suggest that the influence of fetal exposure to PM_2.5_ on childhood BMI trajectories may depend on maternal smoking. We previously reported a main effect association between maternal smoking during pregnancy and rapid BMI growth in early childhood.^4^ This finding is consistent across numerous other studies.^26–28,30–32^ Less is known about the main effect of fetal exposure to PM_2.5_ on childhood BMI trajectories. In the Project Viva cohort, Fleisch et al.^9^ reported no difference in BMI trajectories by PM_2.5_ exposure status. Our interaction results are supported by previous research examining the impact of postnatal exposures on childhood BMI trajectories. In the Southern California Children’s Health Study, McConnell and colleguages^33^ reported that BMI growth from ages 10 to 18 years was most rapid among children with exposure to both secondhand smoke and near roadway pollution. The combination of these exposures during fetal development may impose similar effects on childhood BMI trajectories.

The mechanisms linking fetal exposure to PM_2.5_ and maternal smoking to offspring growth are not yet clear. Both exposures contribute to maternal, placental, or fetal inflammation,^12–14,34^ which is associated with decreased weight and impaired function of the placenta.^35^ Low-grade systemic maternal inflammation can disrupt the regulation of maternal appetite and metabolism, which may have residual effects on offspring growth.^36^ Additionally, these exposures may skew the ratio of white adipose tissue (responsible for storing excess energy) to brown adipose tissue (responsible for dissipating heat)^37^ and alter the metabolic profile of fetal adipose tissue,^38^ a programming effect that may contribute to the risk for adiposity later in life. Finally, the effects of maternal smoking on offspring growth may be exacerbated by contemporaneous exposure to PM_2.5_.^39^ Due to its vasoconstriction properties,^40^ nicotine can induce fetal hypoxia and intrauterine growth restriction,^41^ which may be augmented by further environmental insults.^39^

Our results suggest that the third trimester represents an important developmental window for the programming of offspring growth. The majority of adipose tissue growth occurs in the final few weeks of gestation,^42^ which explains why previous studies report a positive association between increased fetal exposure to PM_2.5_ and low birth weight, based on exposure during the third trimester.^8,16,43–45^ However, contrary to our hypothesis, we did not detect a statistically significant interaction between fetal exposure to maternal smoking and PM_2.5_ on birth weight, although there were some indications of lower neonatal adiposity. Our mixed-effects models indicated that the combined influence of these exposures on BMI increased over time, such that the mean difference in BMI between increased from 0.5 kg/m^2^ at 1 year of age to 1.7 kg/m^2^ by 3 years of age. Therefore, the hypothesized programming effect may not be evident at birth. Future work is needed to identify the windows of susceptibility, which will inform public health opportunities aimed at reducing these exposures among pregnant women.

In this analysis, we did not detect any interactions with O_3_. This may be expected, given the unclear link between fetal exposure to O_3_ exposure and birth weight. Some studies report a positive association between higher exposure to O_3_ and lower birth weight, based on exposure throughout the entire pregnancy^46–49^ or during the third trimester.^5,50^ Other studies, including from our own cohort, have reported no association,^10,11,51,52^ and some have reported that O_3_ may have a slight protective effect against low birth weight^53^ or small for gestational age.^54^

Our study is subject to some limitations. We relied on the maternal residential address reported at enrollment to estimate fetal exposure to O_3_ and PM_2.5_. Our inability to account for potential residential mobility during pregnancy may have contributed to exposure misclassification, resulting in biased results for mothers who did move during pregnancy. However, previous studies have indicated that few women moved during pregnancy.^55,56^ Among those who did move, residential mobility tended to be of short distance and had a minimal impact on exposure assignment.^55,56^ Furthermore, estimating exposure based on residence alone does not account for other microenvironments that may have contributed to exposure, such as at their workplace or while commuting.^57^ Nondifferential error in these measures of exposure may have biased the effect estimates towards the null.^58^

Although we adjusted for individual- and neighborhood-level socioeconomic variables, there remains the possibility for residual confounding by socioeconomic position. Additionally, our study may have been underpowered to detect statistical interactions due to the low number of smokers in our sample (n = 61). Finally, we performed a number of statistical tests. However, given the limited power in our study, we did not adjust our *P* values for multiple testing. Therefore, we acknowledge that our interaction results may be due to chance.

Our use of cotinine is a notable strength of this study. Cotinine is an objective biomarker of nicotine exposure that is considered to be more accurate than maternal self-report of smoking during pregnancy.^59^ Another important strength of our approach is the detailed information about early-life factors that may influence offspring growth, including gestational weight gain, the duration of exclusive breastfeeding, and postnatal exposure to secondhand smoke. These data were not incorporated into the 3 population-based studies which explored the interaction between particulate pollution and maternal smoking on birth weight.^15–17^

## Conclusions

Although PM_2.5_ was generally below the 2012 EPA annual air quality standard of 12.0 μg/m^3^, exposure during the third trimester may influence early-life growth when combined with maternal smoking. These interaction results point to the potential for harmful overloading of environmental insults during pregnancy on offspring growth. Future work in other cohorts may help to further understand the synergistic relationship between these environmental exposures, with the goal of identifying potential interventions that may ameliorate the adverse effects induced by such exposures.

## Supplementary Material


