# Supplementary material for: Joint effects of ambient air pollution and maternal smoking on neonatal adiposity and childhood BMI trajectories in the Healthy Start study
Source: Environ Epidemiol. 2021 May 5;5(3):e142. doi: 10.1097/EE9.0000000000000142 (PMC8196098; doi:10.1097/EE9.0000000000000142)
Supplement: Supplementary file 1 [file ee9-5-e142-s001.docx]

**Supplemental Table S1.** Characteristics of the Healthy Start cohort, the neonatal body composition sample, and the BMI growth trajectories sample

|  | Healthy Start cohort (n=1,410) | Neonatal body composition sample  (n=575) | BMI growth trajectories sample  (n=434) |
| --- | --- | --- | --- |
| Maternal age, years | 28±6 | 29±6 | 29±6 |
| Race/Ethnicity |  |  |  |
| Non-Hispanic white | 53% | 53% | 52% |
| Non-Hispanic black | 15% | 13% | 15% |
| Hispanic | 25% | 27% | 27% |
| Other | 6% | 7% | 6% |
| Household income |  |  |  |
| <$40,000 | 30% | 26% | 24% |
| $40,001 to $70,000 | 20% | 19% | 18% |
| >$70,000 | 32% | 36% | 37% |
| Don’t know | 18% | 18% | 21% |
| Highest level of education |  |  |  |
| <High school | 14% | 14% | 15% |
| High school degree | 18% | 16% | 16% |
| Some college | 67% | 71% | 69% |
| Male offspring | 53% | 51% | 51% |
|  |  |  |  |

Abbreviations: BMI, body mass index
Continuous variables are expressed as means ± standard deviation. Categorical variables are expressed as proportions of column totals.

**Supplemental Table S2.** Predicted BMI values (kg/m^2^) according to fetal exposure to maternal smoking and third trimester PM_2.5_, based on mixed-effects models

|  | Exposure categories^a^ | | | |
| --- | --- | --- | --- | --- |
| Child age | No exposure | High PM_2.5_ only | Maternal smoking only | Both exposures |
| 1 month | 14.4 (14.3, 14.5) | 14.4 (14.2, 14.6) | 14.3 (13.9, 14.7) | 14.2 (13.6, 14.8) |
| 6 months | 15.4 (15.3, 15.5) | 15.4 (15.2, 15.6) | 15.5 (15.1, 15.9) | 15.7 (15.1, 16.3) |
| 12 months | 16.1 (16.0, 16.2) | 16.1 (15.9, 16.3) | 16.3 (15.9, 17.4) | 16.8 (16.2, 17.4) |
| 18 months | 16.6 (16.5, 16.8) | 16.7 (16.5, 16.9) | 17.0 (16.5, 17.4) | 17.6 (16.9, 18.3) |
| 24 months | 17.1 (16.9, 17.2) | 17.1 (16.9, 17.3) | 17.5 (17.0, 18.0) | 18.3 (17.5, 19.1) |
| 30 months | 17.5 (17.3, 17.6) | 17.5 (17.3, 17.8) | 18.0 (17.4, 18.5) | 18.9 (18.1, 19.7) |
| 36 months | 17.8 (17.6, 18.0) | 17.9 (17.6, 18.1) | 18.4 (17.8, 19.0) | 19.5 (18.6, 20.4) |
|  |  |  |  |  |

Abbreviations: BMI, body mass index; PM_2.5_, fine particulate matter.

^a^Exposure categories were defined as follows: no exposure (low PM_2.5_ [between 5.1 and 8.1 μg/m^3^] and cotinine<31.5 ng/mL); high PM_2.5_ only (high PM_2.5_ [between 8.1 and 12.7 μg/m^3^] and cotinine<31.5 ng/mL); maternal smoking only (low PM_2.5_ and cotinine≥31.5 ng/mL); and both exposures (high PM_2.5_ and cotinine≥31.5 ng/mL).

**Supplemental Table S3.** Adjusted beta coefficients and 95% CIs for the association between fetal exposure to maternal smoking and PM_2.5_ with childhood BMI z-score trajectories

| Covariates | Whole pregnancy | Trimester 1 | Trimester 2 | Trimester 3 |
| --- | --- | --- | --- | --- |
| Cotinine (smoker versus non-smoker) | -0.1 (-0.4, 0.3) | -0.1 (-0.4, 0.3) | -0.2 (-0.6, 0.1) | -0.1 (-0.4, 0.2) |
| PM_2.5_ (high versus low) | 0.1 (-0.1, 0.2) | 0.0 (-0.2, 0.2) | 0 (-0.2, 0.1) | 0 (-0.2, 0.2) |
| Age (years) | **0.20 (0.16, 0.23)** | **0.21 (0.16, 0.25)** | **0.19 (0.14, 0.22)** | **0.19 (0.14, 0.23)** |
| Cotinine*PM_2.5_ | -0.3 (-0.8, 0.12) | -0.1 (-0.7, 0.4) | 0.2 (-0.3, 0.8) | -0.1 (-0.6, 0.4) |
| Cotinine*Age | **0.3 (0.2, 0.4)** | **0.2 (0.1, 0.3)** | **0.3 (0.2, 0.4)** | **0.2 (0.1, 0.3)** |
| PM_2.5_*Age | 0.0 (-0.1, 0.1) | 0 (-0.1, 0.1) | 0 (-0.1, 0.1) | 0 (-0.1, 0.1) |
| Cotinine*PM_2.5_*Age | -0.1 (-0.3, 0.1) | 0.1 (-0.2, 0.3) | -0.1 (-0.3, 0.1) | 0.2 (0, 0.5) |
| p for three-way interaction | p=0.43 | p=0.52 | p=0.38 | p=0.09 |

Abbreviations: BMI, body mass index; CI, confidence interval; PM_2.5_, fine particulate matter.

^a^All models adjusted for offspring sex, gestational age at birth (weeks), maternal pre-pregnancy BMI (kg/m^2^), gestational weight gain (kg), maternal education (high school, some college, college), maternal race/ethnicity (non-Hispanic white, non-Hispanic black, Hispanic, other), annual household income (<$40,000, $40,001 to $70,000, >$70,000, missing or do not know), temperature (F), birth year (2010, 2011, 2012, 2013, 2014), season of birth (spring, summer, fall, winter), median household income by Census tract (in $1,000s), household smokers in early childhood (any, none), and the duration exclusive breastfeeding (<5 months, ≥5 months).

^b^The cotinine categories were defined as follows: non-smoker (<31.5 ng/mL) or active smoker (≥31.5 ng/mL).

^c^The PM_2.5_ categories were defined as follows: low (first and second tertile of PM_2.5_) and high (third tertile of PM_2.5_).

**Supplemental Table S4.** Adjusted beta coefficients and 95% CIs for the association between fetal exposure to maternal smoking and O_3_ with childhood BMI z-score trajectories

| Covariates | Whole pregnancy | Trimester 1 | Trimester 2 | Trimester 3 |
| --- | --- | --- | --- | --- |
| Cotinine (smoker versus non-smoker) | -0.2 (-0.5, 0.1) | -0.2 (-0.5, 0.2) | -0.1 (-0.4, 0.2) | -0.2 (-0.5, 0.1) |
| O_3_ (high versus low) | **0.2 (0, 0.4)** | 0 (-0.2, 0.3) | 0 (-0.3, 0.2) | -0.1 (-0.3, 0.2) |
| Age (years) | **0.17 (0.12, 0.21)** | **0.22 (0.18, 0.26)** | **0.18 (0.14, 0.22)** | **0.20 (0.15, 0.23)** |
| Cotinine*O_3_ | 0 (-0.5, 0.5) | 0 (-0.5, 0.5) | -0.2 (-0.7, 0.3) | 0.2 (-0.3, 0.8) |
| Cotinine*Age | **0.2 (0.1, 0.3)** | **0.3 (0.1, 0.4)** | **0.7 (0.2, 1.1)** | **0.3 (0.2, 0.4)** |
| O_3_*Age | **0.11 (0.04, 0.18)** | -0.03 (-0.13, 0) | **0.07 (0, 0.15)** | 0 (-0.1, 0.1) |
| Cotinine*O_3_*Age | 0.1 (-0.1, 0.4) | 0.1 (-0.1, 0.3) | 0 (-0.2, 0.3) | -0.2 (-0.5, 0.1) |
| p for three-way interaction | p=0.28 | p=0.53 | p=0.69 | p=0.10 |

Abbreviations: BMI, body mass index; CI, confidence interval; O_3_, ozone.

^a^All models adjusted for offspring sex, gestational age at birth (weeks), maternal pre-pregnancy BMI (kg/m^2^), gestational weight gain (kg), maternal education (high school, some college, college), maternal race/ethnicity (non-Hispanic white, non-Hispanic black, Hispanic, other), annual household income (<$40,000, $40,001 to $70,000, >$70,000, missing or do not know), temperature (F), birth year (2010, 2011, 2012, 2013, 2014), season of birth (spring, summer, fall, winter), median household income by Census tract (in $1,000s), household smokers in early childhood (any, none), and the duration exclusive breastfeeding (<5 months, ≥5 months).

^b^The cotinine categories were defined as follows: non-smoker (<31.5 ng/mL) or active smoker (≥31.5 ng/mL).

^c^The O_3_ categories were defined as follows: low (first and second tertile of O_3_) and high (third tertile of O_3_).
